# An assessment of the construct validity of the ASCOT measure of social care-related quality of life with older people

**DOI:** 10.1186/1477-7525-10-21

**Published:** 2012-02-10

**Authors:** Juliette N Malley, Ann-Marie Towers, Ann P Netten, John E Brazier, Julien E Forder, Terry Flynn

**Affiliations:** 1 Personal Social Services Research Unit and Quality and Outcomes of Person-Centred Care Research Unit, University of Kent, Canterbury, Kent CT2 7NF, UK; 2 Personal Social Services Research Unit and Quality and Outcomes of Person-Centred Care Research Unit, London School of Economics and Political Science, Houghton Street, London WC2A 2AE, UK; 3 Health Economics and Decision Science, School of Health and Related Research, University of Sheffield, Regent Court, 30 Regent Street, Sheffield S1 4DA, UK; 4 Centre for the Study of Choice (CenSoC), University of Technology Sydney, Sydney, P.O. Box 123, Broadway, NSW 2007, Australia

**Keywords:** social care, quality of life, ASCOT, validity, long-term care

## Abstract

**Background:**

The adult social care outcomes toolkit (ASCOT) includes a preference-weighted measure of social care-related quality of life for use in economic evaluations. ASCOT has eight attributes: personal cleanliness and comfort, food and drink, control over daily life, personal safety, accommodation cleanliness and comfort, social participation and involvement, occupation and dignity. This paper aims to demonstrate the construct validity of the ASCOT attributes.

**Methods:**

A survey of older people receiving publicly-funded home care services was conducted by face-to-face interview in several sites across England. Additional data on variables hypothesised to be related and unrelated to each of the attributes were also collected. Relationships between these variables and the attributes were analysed through chi-squared tests and analysis of variance, as appropriate, to test the construct validity of each attribute.

**Results:**

301 people were interviewed and approximately 10% of responses were given by a proxy respondent. Results suggest that each attribute captured the extent to which respondents exercised choice in how their outcomes were met. There was also evidence for the validity of the control over daily life, occupation, personal cleanliness and comfort, personal safety, accommodation cleanliness and comfort, and social participation and involvement attributes. There was less evidence regarding the validity of the food and drink and dignity attributes, but this may be a consequence of problems finding good data against which to validate these attributes, as well as problems with the distribution of the food and drink item.

**Conclusions:**

This study provides some evidence for the construct validity of the ASCOT attributes and therefore support for ASCOT's use in economic evaluation. It also demonstrated the feasibility of its use among older people, although the need for proxy respondents in some situations suggests that developing a version that is suitable for proxies would be a useful future direction for this work. Validation of the instrument on a sample of younger social care users would also be useful.

## Background

The term social care is used frequently in the UK to describe a range of long-term care activities, including providing help with personal hygiene, dressing and feeding, as well as help with shopping, keeping active and socialising. Social care services include personal assistance, nursing and residential care homes, and day centres and are usually provided in response to needs arising from physical or sensory impairments, learning difficulties and mental health problems including dementia [1]. The increased cost of this support, associated with improvements in life expectancy among disabled people and in the general population, continues to be the subject of much analysis and debate [2-5]. If we are to target resources effectively we also need to be able to measure the outcomes and value of such services.

Measuring outcomes is never straightforward but is arguably particularly challenging in social care due to the nature of the support provided. Social care services tend to compensate people for the effect of their impairments on their quality of life (QoL) in accordance with local and national policies. Services also aim to do this in a way that is enabling and allows people to make choices as to how their needs are met. If we want to measure the value of social care services we need a measure that reflects the compensatory activity of social care, is sensitive to choice and captures what we have termed *social care-related quality of life *(SCRQoL), which reflects those aspects of QoL, or attributes, that are the focus of social care support. Finally to generate a single score for use in cost-effectiveness analyses it should be preference-weighted to reflect the relative importance of the SCRQoL states [6].

The ASCOT instrument was developed as a multi-attribute preference-weighted measure of SCRQoL. ASCOT has eight conceptually distinct attributes: *Personal cleanliness and comfort, Food and drink, Control over daily life, Personal safety, Accommodation cleanliness and comfort, Social participation and involvement, Occupation *and *Dignity. Dignity *differs from the other attributes since it reflects the impact of the care process on how people feel about themselves, rather than being an aspect of QoL that applies to all of us, whether or not we have care and support from others. There is one item per attribute and each attribute has four response options, reflecting four different outcome states. The top two states both reflect states where outcomes are fully realised but were designed to differ in the extent to which respondents have choice over how the outcome is realised, so that the best state reflects a person with choice and the second state one without choice [6]. To assess the distribution of responses to the ASCOT attributes, a survey was conducted which also afforded an opportunity to assess the validity of the measure.

Validity is an assessment of the extent to which the instrument measures what it is intended to represent, which for the ASCOT measure is the value of social care. A number of different types of validity have been identified, including content, construct, and criterion-related validity [7]. However, the psychometric approach used to assess validity needs some modification to make it applicable for determining the validity of preference-weighted measures, which capture the value of the outcome state not the state itself [8,9]. Brazier et al [9] identify three aspects of preference-weighted measures that require validation: the descriptive system, the valuations, and the empirical validity of the instrument. The valuations derived from the preference study have been examined elsewhere [6,10]. The focus of this paper is on the validity of the descriptive system, which refers to the choice of attributes and the specification of the items in the instrument. Since there is a lack of well-established measures for many of the ASCOT attributes, our approach to validation follows that taken by Coast et al [11], and focuses on the construct validity of each of the attributes: the extent to which each attribute has the expected relationship with other variables and concepts. We first describe the method before discussing the study results.

## Methods

The data were collected to assess the distribution of responses to ASCOT and explore its validity. Ethics approval was obtained from the University of Kent Ethics Committee and research governance approval from each of the Local Authorities (LAs) that agreed to participate in the study. A sampling frame was generated from respondents to a national user experience survey (UES) of older people (aged over 65) using home care services who had indicated that they were happy to be approached to take part in further research [12].

Data were collected face-to-face through computer-aided personal interviews in people's homes during 2009 in ten geographically dispersed locations across England. The interviews gathered socio-demographic information and details about service receipt and informal support. To measure health we used the five-dimension Euroqol (EQ-5D) [13,14]; a range of activities of daily living (ADLs) and instrumental activities of daily living (IADLs) were included to measure disability; and the 12-question General Health Questionnaire (GHQ-12)[15-17] was included to capture psychological well-being. The control and autonomy subscale of the CASP-12 [18,19] was used to measure sense of control. To capture QoL a global self-reported seven-point measure of QoL was included. Four measures of aspects of the nature of the locality and environment were included: a five-point interviewer-rated cleanliness and tidiness of respondent's home, the type of area the person lived in (rural, urban and so on), a four-point self-rated design of home question [20] and a four-point question on the accessibility of local area. Measures of social contact and participation included: the UCLA 3-item loneliness scale [21], and measures on the frequency of meeting up with friends and relatives, the frequency of speaking to neighbours, the frequency of speaking to friends and relatives, involvement in organised formal and informal groups and activities in the last 12 months, and unpaid volunteering in last 12 months. Permission was gained to link the interview data to that from the UES, conducted roughly six months previously, which contained questions on perceptions of service quality.

We used hypothesis testing to test construct validity for each ASCOT attribute. Expected relationships with variables were based on evidence from the literature, where this was available, and the views of team members, who brought their expertise in this area, as well as detailed knowledge of the data from which ASCOT items were developed. Table 1 summarises the hypothesised associations for each attribute. Relationships were analysed between all the ASCOT attributes and each variable for transparency and counterfactual evidence.

**Table 1:** Variables included and expected associations

*Variables*	*Expected associations*
***Socio-demographics***	

Marital status and living situation	We expect people with a partner or those living with others to feel safer and expected an association with *Safety *[25]. We also expected a positive association with *Social participation *as co-residents provide a source of social contact and stimulation.

***Well-being***	

Global 7-point self-rated QoL	Since all ASCOT attributes are aspects of QoL, positive associations were expected for each attribute with global self-rated QoL.
GHQ-12 [15-17]	GHQ-12 was used as an indicator of psychological well-being. Research has demonstrated the relationship between psychological and emotional well-being and QoL, so we expected positive associations with all attributes [36,37].

***Health and disability***	

EQ-5D [13,14]	There is a close relationship between health and QoL, so we expected positive associations with all attributes [36-39].
ADLs and IADLs	ADLs and IADLs are frequently used as measures of 'need' in social care research since they capture how well the individual functions in their daily life unaided. They do not capture the compensatory activity of social care. However, since these measures capture restrictions in a person's ability to manage independently, we would expect positive associations with *Control*. We also expected a positive relationship between the personal care ADLs and *Personal cleanliness*, and the food-related ADLs and IADLs and *Food and *drink, but with the relationship restricted to those reporting they could manage on their own being more likely to choose the top level.

***Control and autonomy***	

Control and autonomy subscale of CASP-12 [18,19]	We anticipated a positive association between this measure and *Control*. We also expected to find differences in subscale scores between the top and second response option for each attribute, reflecting the sense of choice conveyed by the wording of the top level.

***Nature of locality and environment***	

5-point interviewer-rated cleanliness and tidiness of respondent's home	We expected interviewer judgements of cleanliness and tidiness of the home to be positively associated with *Accommodation*.
4-point self-rated design of home [20]	A poorly-designed home will make it more difficult for optimal care to be provided in the home [40,41], so we anticipated positive associations with *Control, Personal cleanliness, Accommodation *and *Safety *attributes.

Type of area (London, city/large town, suburb of city/large town, small town, rural/village)	We anticipated that area would be associated with *Safety*, as people living in more urban areas are likely to feel less safe due to fears about crime [25,42,43]. We also expected urban areas to be associated with better outcomes in the *Occupation *and *Social participation *attributes due to better transport links and amenities.
4-point self-rated accessibility of local area	We expected poor accessibility of the local area to be a barrier to achievement of outcomes in the *Control, Occupation *and *Social participation *attributes.

***Social contact and support***	

Frequency of meeting up with friends and relatives, frequency of speaking to neighbours, frequency of speaking to friends and relatives UCLA 3-item loneliness scale [21].	Measures of contact with people outside of the home were expected to be strongly positively associated with *Social participation*. We also expected a positive association with *Occupation *because social activities are a way of occupying one's time. In addition, a positive association with *Safety *was hypothesised since supportive networks are likely to enhance a person's sense of safety [23,25]. A negative association with *Social participation *was expected as loneliness has consistently been shown to be related to social contact [44,45]. A negative association with *Safety *is likely due to people who feel lonely being more likely to feel vulnerable [25]. Negative associations with all the other attributes, except *Dignity*, are likely to be observed as people who are lonely are likely to lack support to achieve good outcomes and loneliness is closely associated with depression [46], which has been shown to be related to poor QoL [37].

***Participation***	

Involvement in organised formal and informal groups and activities in last 12 months, unpaid volunteering in last 12 months	We anticipated a positive association between taking part in groups and volunteering and *Occupation*, in particular. A positive association with *Social participation *was also expected as organised groups are a source of social contact.

***Service quality***	

Items capturing the quality of care delivery by care workers (see same care workers, come at suitable times, do the things you want done, arrive on time, in a rush, spend less time than supposed to, informed about changes in your care, global rating of way treated by care workers) [27]	A positive association was expected between these items capturing aspects associated with the quality of the delivery of care by care workers and *Dignity *since *Dignity *is included in the measure to capture the effect of the way care is delivered on a person's sense of self-worth. In particular, we expected the global rating of the way the person felt they were treated by the care worker to be associated with *Dignity*.

To assess relationships, we used chi-squared tests (for unordered or ordered categorical variables) or one-way analysis of variance (for continuous variables) in STATA v11. For chi-squared tests, one-sided probability exact tests were used when computationally feasible; where this was not possible, data were recoded to increase numbers in individual cells. Fisher's exact test produces a p-value indicating the probability that the two variables are independent of one another. Unlike chi-squared, there are no accompanying test statistics. Consequently, the p-values only are summarised in the tables and the nature and direction of the associations are reported in the text. Associations significant at the 1% level were taken to be strongly suggestive of a relationship between the attribute and the variable; and relationships significant at between the 1% and 10% level were taken to be weakly suggestive of a relationship between the attribute and the variable. We also considered the patterns of relationships as well as the p-value to assess the direction of the relationships rather than significance alone.

## Results

In total, 566 contacts were attempted from a sample of 778, producing 301 (53%) complete interviews. Non-responders were categorised as refusals (n = 18, 3%), deceased (n = 4, 1%) and not contactable (n = 243, 43%). Table 2 shows the characteristics of the 301 participants. As expected among publicly-funded older home care service users, the majority of the sample was female, over the age of 80, single and lived alone [3,12]. However, compared with the population from which this sample was drawn, the sample has slightly fewer females (68% compared with 72%), more people from a white ethnic background (98% compared with 93%) and a smaller proportion over 80 years old (60% compared with 68%) [12].

**Table 2:** Socio-demographic and economic characteristics of sample members

	*Frequency*	*Percentage*
*Sex (n = 301)*		
Female	205	68.1%

*Ethnicity (n = 296)*		
White	296	98.3%

*Age (n = 301)*		
65 to 69	27	9.0%
70 to 79	95	31.6%
80 to 89	137	45.5%
90 and above	42	14.0%

*Area live in (n = 301)*		
London borough	35	11.6%
Another large city or town	59	19.6%
Suburbs of large city/town	47	15.6%
Small town	98	32.6%
Rural area or village	62	20.6%

*Marital status (n = 301)*		
Married/living together	82	27.2%
Never-married	30	10.0%
Widowed	168	55.8%
Separated/divorced	19	6.3%

*Living situation (n = 301)*		
Live alone	202	67.1%

*Tenure (n = 301)*		
Owner-occupier	154	51.2%
Rent-paying tenant	142	47.2%
Tenant living rent free	5	1.7%

*Income (n = 182)*		
£275 or less per week	122	40.5%
£276-374	40	13.3%
£375-424	13	4.3%
£425-£574	2	0.7%
£575 per week or more	5	1.7%

The distribution of non-proxy responses for each ASCOT attribute is shown in Table 3 along with the proportion of responses answered by a proxy. Ten per cent or fewer of the responses to ASCOT attributes were given by a proxy. This was very similar to the percentages for the EQ-5D, GHQ-12, ADL and QoL question, with proxy response rates ranging from 8.6% to 10.6%.

**Table 3:** Responses of older people receiving home care to the ASCOT questionnaire (n = 301)

*Attribute*	*Frequency (Percent)*	*Number answered by proxy (Percent)*
*Control over daily life (n = 270)*		31 (10.3)
I have as much control over my daily life as I want	87 (32.2)	
I have adequate control over my daily life	86 (31.9)	
I have some control over my daily life	82 (30.4)	
I have no control over my daily life	15 (5.6)	

*Personal cleanliness and comfort (n = 273)*		28 (9.3)
I feel clean and am able to present myself the way I like	165 (60.4)	
I feel adequately clean and presentable	87 (31.9)	
I feel less than adequately clean or presentable	19 (7.0)	
I don't feel at all clean or presentable	2 (1.0)	

*Food and drink (n = 271)*		30 (10.0)
I get all the food and drink I like when I want	213 (78.6)	
I get food and drink adequate for my needs	55 (20.3)	
I don't get all the food and drink I need, but I don't think there is a risk to my health	1 (0.4)	
I don't get all the food and drink I need, and I think there is a risk to my health	2 (0.7)	

*Accommodation cleanliness and comfort (n = 272)*		29 (9.6)
My home is as clean and comfortable as I want	173 (63.6)	
My home is adequately clean and comfortable	92 (33.8)	
My home is less than adequately clean and comfortable	6 (2.2)	
My home is not at all clean and comfortable	1 (0.4)	

*Safety (n = 273)*		28 (9.3)
I feel as safe as I want	154 (56.4)	
Generally I feel adequately safe, but not as safe as I'd like	85 (31.1)	
I feel less than adequately safe	27 (9.9)	
I don't feel at all safe	7 (2.6)	

*Social participation (n = 271)*		30 (10.0)
I have as much social contact as I want with people I like	105 (38.8)	
I have adequate social contact with people	94 (34.7)	
I have some social contact with people, but not enough	55 (20.3)	
I have little social contact with people and feel socially isolated	17 (6.3)	

*Occupation (n = 272)*		29 (9.6)
I'm able to spend my time as I want, doing things I value or enjoy	85 (31.3)	
I'm able to do enough of the things I value or enjoy with my time	72 (26.5)	
I do some of the things I value or enjoy with my time but not enough	103 (37.9)	
I don't do anything I value or enjoy with my time	12 (4.4)	

*Dignity (n = 273)*		28 (9.3)
The way I'm helped and treated makes me think and feel better about myself	134 (49.1)	
The way I'm helped and treated does not affect the way I think or feel about myself	106 (38.8)	
The way I'm helped and treated sometimes undermines the way I think or feel about myself	30 (11.0)	
The way I'm helped and treated completely undermines the way I think or feel about myself	3 (1.1)	

We hypothesised that a number of measures -- GHQ-12, overall QoL, EQ-5D, and CASP control and autonomy subscale -- would be associated with all of the attributes and that the UCLA loneliness scale would be associated with all the attributes except *Dignity*. In general, associations significant at the 1% level (p < .01) were found between these measures and all of the attributes (see Table 4), except for the relationships between the CASP subscale and *Food and Drink *and *Dignity*, and the loneliness scale and *Food and Drink *and *Accommodation*, where associations reached the 5% (p < .05) or 10% level (p < .10) of significance.

**Table 4:** Mean GHQ-12, CASP subscale, EQ-5D and UCLA loneliness scale scores by ASCOT attribute

*Attribute*	*GHQ-12 (N)*	*CASP subscale (N)*	***EQ-5D***^***1***^***(N)***	*UCLA Loneliness (N)*
*Control over daily life*				
I have as much control over my daily life as I want	25.98 (87)	11.98 (87)	0.43 (87)	1.09 (87)
I have adequate control over my daily life	23.71 (86)	9.84 (86)	0.35 (86)	2.29 (86)
I have some control over my daily life	21.10 (78)	8.49 (80)	0.18 (81)	2.73 (80)
I have no control over my daily life	15.62 (13)	6.36 (14)	-0.11 (14)	2.93(14)
F statistic and significance	17.69***	20.78***	14.71***	12.48***

*Personal cleanliness and comfort*				
I feel clean and am able to present myself the way I like	25.02 (159)	10.90 (161)	0.37 (162)	1.63 (161)
I feel adequately clean and presentable	21.60 (85)	8.79 (86)	0.24 (85)	2.62 (86)
I feel less than adequately clean and presentable	18.16 (19)	7.00 (19)	0.06 (19)	3.16 (19)
I don't feel at all clean or presentable	7.50 (2)	6.00 (2)	-0.21 (2)	4.00 (2)
F statistic and significance	16.90***	12.43***	7.42***	7.92***

*Food and drink*				
I get all the food and drink I like when I want	24.23 (208)	10.27 (211)	0.34 (212)	1.89 (210)
I get food and drink adequate for my needs	19.87 (53)	8.74 (54)	0.14 (54)	2.70 (54)
I don't get all the food and drink I need⍰	20.00 (3)	8.67 (3)	-0.04 (3)	2.33 (3)
F statistic and significance	11.23***	3.89**	7.95***	3.67**

*Accommodation cleanliness and comfort*				
My home is as clean and comfortable as I want	24.81 (169)	10.65 (171)	0.36 (172)	1.79 (170)
My home is adequately clean and comfortable	20.90 (88)	8.68 (90)	0.19 (90)	2.58 (90)
My home is less than adequately clean and comfortable/My home is not at all clean and comfortable⍰	17.43 (7)	8.14 (7)	0.10 (7)	2.71 (7)
F statistic and significance	15.74***	9.68***	8.21***	5.12**

*Safety*				
I feel as safe as I want	24.8 (152)	10.76 (153)	0.33 153)	1.71 (153)
I feel adequately safe, but not as safe as I'd like	23.21 (81)	9.38 (82)	0.34(83)	2.01 (82)
I feel less than adequately safe	17.73 (26)	7.44 (27)	0.10 (27)	3.74 (27)
I don't feel at all safe	13.14 (7)	8.43 (7)	-0.17 (7)	4.14 (7)
F statistic and significance	18.89***	8.10***	7.64***	11.79***

*Social participation*				
I have as much social contact as I want with people I like	25.69 (105)	11.68 (105)	0.36 (105)	1.05 (105)
I have adequate social contact	23.21 (90)	9.52 (93)	0.33 (94)	2.12 (92)
I have some social contact with people, but not enough	20.39 (54)	8.18 (55)	0.17 (55)	3.38 (55)
I have little social contact with people and feel socially isolated	18.82 (17)	7.06 (17)	0.17 (17)	3.71 (17)
F statistic and significance	13.27***	18.48***	4.07***	27.04***

*Occupation*				
I'm able to spend my time as I want, doing things I value or enjoy	26.61 (85)	12.35 (85)	0.44 (85)	1.21 (85)
I'm able to do enough of the things i value or enjoy with my time	23.54 (72)	10.07 (72)	0.30 (72)	1.86 (72)
I do some of the things I value/enjoy with my time but not enough	20.90 (98)	8.09 (102)	0.20 (102)	2.77 (101)
I don't do anything I value or enjoy with my time	18.36 (11)	7.00 (11)	0.07 (12)	3.64 (11)
F statistic and significance	17.52***	30.20***	9.14***	13.67***

*Dignity*				
The way I'm helped and treated makes me think and feel better about myself	24.17 (132)	9.99 (132)	0.34 (133)	1.97 (132)
The way I'm helped and treated does not affect the way I think/feel about myself	23.52 (105)	10.40 (106)	0.34 (106)	2.10 (106)
The way I'm helped and treated sometimes undermines the way I think/feel about myself	19.44 (27)	8.31 (29)	-0.00 (29)	2.85 (29)
The way I'm helped and treated completely undermines the way I think/feel about myself	16.33 (3)	8.33 (3)	0.23 (3)	3.33 (3)
F statistic and significance	5.74***	2.63*	7.94***	2.09

^1^Time trade-off weighted scores used

⍰ Lowest two levels of the attribute are collapsed because of small numbers

*** significant at 1% level, ** significant at 5% level, * significant at 10% level

The relationship between these measures and the attributes were also in the expected direction - positive for GHQ-12, overall QoL, EQ-5D, and CASP subscale and negative for the loneliness scale (see Table 4). Post-hoc tests showed a significant difference in average CASP subscale scores between the top and second response options for all of the attributes except *Dignity*. For *Dignity *a significant difference was found between the responses to the second response option and the options that report feeling undermined.

For both the GHQ-12 and EQ-5D, post-hoc tests showed a significant difference in average scores between the top and second response options for all attributes, except *Dignity, Safety *and *Social participation *(for EQ-5D only) where the difference was between the top two and the bottom two options. Average scores were also significantly different for each level of *Control*. Post-hoc tests for the loneliness scale revealed that people reporting the top level were significantly less lonely compared to those reporting worse outcomes, for all attributes except *Safety *and *Occupation *where those reporting the top two levels were significantly less lonely than those choosing the bottom two levels, and *Dignity *where no significant differences were found.

Results for variables hypothesised to be related to each attribute are summarised below. The significance of the relationships between the measures of socio-demographic characteristics, disability, nature of the locality and environment, social contact and support, participation and service quality and the eight attributes are summarised in Tables 5 and 6 and the nature and direction of the associations are reported in the text below.

**Table 5:** Significance of relationship between ASCOT attributes and socio-demographic characteristics, general quality of life and disability (p-values)

	*Control*	*Personal Cleanliness and comfort*	*Food and drink*	*Accomm cleanliness and comfort*	*Safety*	*Social participation*	*Occupation*	*Dignity*
***Socio-demographic characteristics***								
Marital status^a^	.037**	.199	.039**	.081*	.074*	.574	.028**	.106
Live alone^a^	.004***	.552	.019**	.599	.029**	.967	.210	.930

***Quality of Life***								
Quality of Life^b^	< .001***	< .001***	.007***	< .001***	< .001***	< .001***	< .001***	.004***

***Disability: activities of daily living and instrumental activities of daily living***								
Stairs^a^	.047**	.170	.617	.059*	.808	.254	.119	.397
Outdoors & walk down road^a^	< .001***^b^	.085*	.826	.036**	.638	.374	.006***	.185
Get around indoors^a^	< .001***^b^	.001***	.011**	.026**	.470	.356	.023**	.293
Get in/out bed^a^	< .001***^b^	.002***	.209	.005***	.540	.195	.002***	.254
WC/toilet^a^	< .001***	< .001***	.002***	.010***	.090*	.298	< .001***	.066*
Wash face hands^a^	< .001***	< .001***	.086*	.007***	.188	.662	< .001***	.745
Bath, shower, wash all over^a^	< .001***	< .001***	.622	.046**	.348	.147	< .001***	.451
Dressed/undressed^a^	< .001***	.014**	.931	.453	.738	.936	.113	.631
Feed self^a^	.037**	.008***	< .001***	.277	.007***	.742	.562	.078*
Paperwork/finances^a^	< .001***	.005***	.124	.015**	.012**	.021**	< .001***	.054*
Household shopping^a^	.005***	.162	.611	.196	.001***	.585	.077*	.980
Prepare hot meals^a^	.017**	.028**	.035**	.516	.259	.619	.490	.404

^a ^Fischer's exact test; ^b ^Lowest two levels of the attribute are collapsed, except for *Food and drink *and *Accommodation *where the attribute is dichotomised by collapsing lowest three levels; *** significant at 1% level, ** significant at 5% level, * significant at 10% level

**Table 6:** Significance of relationship between ASCOT attributes and nature of locality and environment, social contact and support and participation (p-values)

	*Control*	*Personal Cleanliness and comfort*	*Food and drink*	*Accomm cleanliness and comfort*	*Safety*	*Social participation*	*Occupation*	*Dignity*
*Nature of locality and environment*								
Interviewer assessed cleanliness/tidiness of home^a^	.702	.014**	.014**	< .001***	.015**	.004***^b^	.503	.515
Design of home^a^	.018**	.005***	.038**	< .001***	.001***	.007***	.075*	.001***
Living area^a^	.075*	.003***	.599	.003***	.011**	.074*	.014***	.004***
Getting around local area^a^	< .001***	.108	.052*	.129	.001***	.012**	< .001***	.024**

*Social contact and support *								
Speak to relatives/friends^b^	.004***	.442	.014**	.118	< .001***	.011**	.003***	.045**
Speak to neighbours^b^	.005***^b^	.006***	.477	.328	.064*^b^	.017***^b^	.005***^b^	.343
Meet up with relatives/friends not living with^b^	.156^b^	.231	.249	.062*	.261	.001***^b^	.003***^b^	.068*

*Participation*								
Taken part in groups last 12 months^a^	.054*	.003***	.231	.512	.180	.125	.003***	.591
Taken part in voluntary work in last 12 months^a^	.084*	.602	.337	.119	.621	.014**	< 001***	.329

^a ^Fischer's exact test; ^b ^Lowest two levels of the attribute are collapsed, except for *Food and drink *and *Accommodation *where the attribute is dichotomised by collapsing lowest three levels

*** significant at 1% level, ** significant at 5% level, * significant at 10% level

### Control over daily life

*Control *was significantly associated with the CASP subscale (p < .001) and, in comparison with the other attributes for which a difference in average scores between the top two response options was found, post-hoc tests revealed that the difference between the mean CASP scores of each *Control *state, except that between the lowest two levels, was significant (see Table 5 for means).

Several measures - ADLs, IADLs, home design and accessibility of local area - captured aspects of one's life that may restrict one's ability to manage independently. We therefore expected them to be associated with *Control*. A positive relationship was observed with home design and accessibility of local area, although the relationship with home design was weaker (p < .05). All of the ADLs (except ability to manage stairs and feed oneself) and IADLs (except prepare hot meals) were significantly (p < .001) associated with *Control *in the manner expected, with people reporting that they could manage the activity on their own more likely to report the top level. For the most part, poorer outcomes were related to whether they managed with difficulty, needed help or did not do the activity. An exception was that difficulty performing the mobility ADLs was not associated with poorer outcomes.

*Control *was unexpectedly associated with living status (p < .001), with those reporting living with others experiencing less control over their daily lives. We also did not anticipate the positive association found with speaking to relatives and speaking to neighbours (p < .001).

### Personal cleanliness and comfort

Although the *Personal cleanliness *item captures the compensatory action of services, we did expect the attribute to be associated with ADLs related to personal care, with those reporting they could manage the personal care ADLs on their own being more likely to choose the top response option. This pattern was observed, and significant associations were found for getting in and out of bed, going to the WC/toilet, washing face and hands, bathing (all p < .001) and getting dressed and undressed (p < .05). Unexpectedly, highly significant associations were also found for getting around indoors and feeding (p < .001), as well as for the IADL managing paperwork and finances (p < .001). A positive association was also observed between home design and *Personal cleanliness *(p < .01), as we hypothesised.

*Personal cleanliness *had an unexpected positive association with participation in groups and speaking to neighbours; there was no association with speaking and meeting up with relatives and friends. Another unanticipated association was that people living in London or another large city or town were more likely to report the top level, with people living in suburban areas and small towns (not rural areas) appearing worst off, both being more likely than expected to choose the lower two levels.

### Food and drink

*Food and drink *was significantly associated with the 'feed self' ADL (p < .001), with those reporting that they could manage on their own being more likely to choose the top level, as we expected. Although in the anticipated direction, with people reporting that they can manage the activity on their own more likely to report the top level, the association with the IADL preparing hot meals was only significant at the 5% level (p < .05); and there was no association with the household shopping IADL. Again, reflecting the effect of home design on outcomes, a positive relationship was observed between this variable and *Food and drink *(p < .05).

*Food and drink *also had a highly significant relationship (p < .001) with using the WC/toilet ADL, with those reporting that they could manage on their own being more likely to choose the top level.

### Accommodation cleanliness and comfort

We hypothesised that *Accommodation *would have a significant, positive association with the interviewer's assessment of the cleanliness and tidiness of the respondent's home and with home design. Both of these relationships were observed (p < .001). *Accommodation *had strong relationships (p < .01) with several ADLs (getting into and out of bed, using the WC/toilet, and washing face and hands), with those reporting that they could manage on their own being more likely to choose the top level. It also had an unexpected relationship with the type of area the person lives in (p < .01), with people living in London or another large city or town more likely to report the top level. People living in suburban areas and small towns (not rural areas) appeared worst off, both being more likely than expected to report the lower two levels.

### Safety

*Safety *was, as we anticipated, positively associated with home design (p < .01). It was also associated with the type of area the respondent lives in (p = .011), with people living in London or another large city or town being more likely than expected to report the lower two levels; people living in suburban areas being more likely than expected to report the third level; and people in small towns and rural areas being more likely than expected to report the second level.

As expected, sense of *Safety *was weakly related to living alone (p < .05) and marital status (p < .10), with those who live with others or are married reporting better outcomes. Both items capturing the extent to which the person speaks to others (to relatives/friends and neighbours) were positively associated with safety, although for speaking to neighbours the association was weak (p < .10). There was no association with meeting up with relatives or friends.

An unexpectedly significant, positive association was also found between *Safety *and people's perceptions of how easy it is to get to all the places in their local area (p = .001). A highly significant association was found with the household shopping IADL (p < .001) and the feed self ADL (p < .01), where people reporting that they can manage the activity on their own were more likely to report the top outcome state.

### Social participation

*Social participation *was significantly associated with the loneliness scale and, unlike the pattern observed for the other attributes, post-hoc tests revealed a significant difference in the average score between each *Social participation *level (see Table 4). In addition, *Social participation *had positive associations, with the social contact and support items (frequency of speaking to relative or friends (p = .011), to neighbours (p = .017), and meeting up with relatives or friends (p = .001)) and having volunteered (p < .05). *Social participation *was not associated with marital status or living alone.

As hypothesised, there was a positive association with accessibility of the local area (p < .05) and a marginally significant association with the type of area the respondent lived in (p < .10): people living in London or another large city were more likely to report good outcomes and those in suburban areas were more likely to report the lower two levels.

*Social participation *was also strongly, positively associated with self-reported design of home (p < .01) and the interviewer's view of the cleanliness and tidiness of the respondent's home (p < .01).

### Occupation

Key anticipated relationships for *Occupation *were found with participation in groups (p = .01) and volunteering (p = .001). In addition, *Occupation *was strongly positively associated with the three social contact and support items (frequency of speaking to relative or friends, to neighbours, and meeting up with relatives or friends (all p < .01)).

As expected, there was an association with the area people lived in (p = .014), with people living in London or another large city or town being more likely to report the top level of *Occupation*. However, it was people living in suburban areas and small towns (not rural areas) who appeared worst off, both being more likely than expected to report the lower levels [22]. *Occupation *also had a significant, positive association with people's perceptions of how easy it is to get to around their local area (p < .001), as hypothesised.

*Occupation *was significantly associated with nearly all the ADL items (see Table 5), with people reporting that they could manage the activity on their own more likely to report the top level. For the most part, poorer outcomes were related to whether the person managed with difficulty, needed help or did not do the activity. An exception to this pattern was that for people reporting difficulty performing the ADLs relating to mobility and using the toilet/WC this was not associated with poorer outcomes. There was also a highly significant association with the managing paperwork and finances IADL (p < .001), with people reporting that they can manage the activity on their own more likely to report the top level.

### Dignity

*Dignity *was significantly associated with the question asking whether care workers do the things the user wants done (p < .001). It was also weakly associated with all of the other user-reported service quality variables, except the question about whether the person sees the same care workers, with p-values ranging from 0.028 to 0.064, such that better quality services were associated with better dignity scores. Unexpectedly, no relationship was found (p = .71) with the question asking users to assess their overall treatment by care workers.

An unanticipated association was found with home design (p < .01), with those reporting that how they are helped makes them think and feel better about themselves being the only group more likely than expected to think the design of their home meets all their needs. A significant and unexplained association was also found with the area people live in (p < .01), with those living in London, another large city or town and suburban areas being less likely than expected to choose the top level and more likely to choose the second response option. By contrast, people living in small towns were more likely to choose the top level and less likely to choose the second level.

## Discussion

The analyses presented in this paper provide some evidence to support the construct validity of the ASCOT attributes. The difference between the top two levels in CASP control and autonomy subscale scores for each attribute provides good evidence of the ability of the items to capture choice. Although for *Dignity *a difference was found between the top two and bottom two levels, when cognitively testing this item we found that those choosing the second response option generally had less need for help and received fewer services, which implies, as we found here, that this group are more independent and less likely to experience loss of control compared to those choosing other response categories.

In terms of the attributes, we have very strong evidence to support the validity of the *Control *and *Occupation *items, in particular, since these had strong associations with the expected variables and where there were unexpected relationships these could be explained. For instance, it is possible that the association between higher levels of *Control *and living with others is related to higher levels of impairment among service users who live with others, as eligibility criteria mean that those living alone are more likely to receive services as they have less access to informal support. In support of this, we found that respondents living with others (U = 4765.50, z = -7.38, p < .001) needed help with significantly more ADLs than people living alone. It is also possible that those with more control (and therefore independence) are able to maintain relationships more easily, hence the positive association between *Control *and speaking to relatives and neighbours [23]. Equally the unexpected finding that it was those living in suburban areas and small towns whom had the worst *Occupation *outcomes, may be explained by suburban areas lacking a sense of community that is strong in rural areas [22]. The association between *Occupation *and the ADLs could be due to the loss of independence associated with difficulty or inability to perform ADLs or IADLs and the restriction this places on the activities a person can enjoy.

There was also very good evidence concerning the *Personal cleanliness *and *Accommodation *attributes, since both had strong associations in the expected direction with key variables. We were not able to understand well the reason for the relationship of these items with the type of area the person lived in. It may be that this is due to LA policies or a quirk of the sample, and validation on a different sample would provide contrary evidence. However, other unanticipated relationships such as the one between *Personal cleanliness *and the non-personal care ADLs and IADLs, is likely to be due to similarities in the capabilities required for the tasks. The positive association of *Personal cleanliness *with speaking to neighbours and participation in groups could reflect an unwillingness of people who do not feel presentable to socialise with people they do not know well [24].

In general, *Social participation *had the anticipated relationships with other variables, although sometimes the associations were weak. The only expected findings that were not observed was an association with marital status and living with others, which suggests that the people in this sample, at least, did not consider those they live with as being a source of social contact. The unexpected strong positive associations with design of home and the interviewer's view of the cleanliness and tidiness of the respondent's home, may reflect an unwillingness amongst people with inaccessible, unclean or untidy homes to accept guests [24].

Similarly, *Safety *seemed to capture factors both inside and outside the home that could make a person feel unsafe, but the lack of association with the frequency with which the person met up with relatives and friends was unexpected, particularly given the associations that were found with speaking to relatives, friends and neighbours. This implies that a sense of safety is determined more by the sense that there is someone to turn to or perhaps a sense of connection to a community, as has been found in research elsewhere [23,25,26] rather than regularly meeting up with friends and relatives. This potentially has implications for the nature of social care support that aims to help people feel safe. The unanticipated relationships with variables capturing the ability of the person to get around outdoors may be explained by fear of falling.

The attributes for which we had the weakest evidence of validity were *Food and drink *and *Dignity. Food and drink *is not easy to validate as good, easy-to-administer, self-reported measures of nutritional intake are lacking. The few measures we did use in general had the expected relationship with the item, but often the relationships were weak. The lack of relationship with household shopping is not too concerning since this could include shopping for items other than food. As we discuss below, the poor distribution of *Food and drink *which resulted in a lack of variation may have affected the analysis undertaken here and it would be of value to repeat the analysis conducted here with the new item wording.

Similarly, we lacked good data against which to validate *Dignity *since the variables that we hypothesised would be strongly associated with *Dignity *-- the variables capturing aspects of quality associated with the delivery of home care services by care workers and related staff - were collected roughly six months prior to the survey data. In general *Dignity *had the expected relationships with other variables, but the associations were mostly weak. The lack of relationship with the question about overall treatment by care workers was unexpected, but given the other significant, albeit weak, relationships, and previous evidence that this question is poorly related to service users' attitudes towards their carers [27], we feel the observed lack of association raises more questions about the overall treatment question rather than the *Dignity *item. The weak associations between *Dignity *and most of the service quality questions could reasonably be explained by the gap of six months between the collection of both sets of data. Differences in breadth of the questions could also be a factor, since the questions about service quality focus on care workers, whereas *Dignity *is broader, asking about help and treatment by any person. It would be of value to repeat this analysis with better validation data.

In addition to the evidence presented here, the methods used to develop the measure ensured content and face validity. Expert review with social care stakeholders was used to identify attributes and ensure ASCOT's sensitivity to outcomes of interest to policymakers and relevance to the evaluation of social care interventions. This approach was complemented by a literature review exploring service users' understanding of social care outcomes. Cognitive interviews were conducted to check social care service users' understanding of terms and clarify the wording of the items [6].

ASCOT was developed to fill a gap, as to our knowledge there are no dedicated social care outcome measures. Past studies in the social care field have tended to use health outcome measures, such as the EQ-5D, to assess cost-effectiveness. Whilst these measures share some of the characteristics of ASCOT, they tend to focus on people's functional abilities (such as mobility) rather than the impact of support on their QoL and are limited in the range of outcome states they capture [28,29]. ACSOT was proposed as a measure to capture the full range of social care outcomes and we would expect it to be a more sensitive measure than the EQ-5D. Early findings suggest that the two measures are strongly correlated (r = 0.4), but that ASCOT is more sensitive than the EQ-5D to the impact of social care interventions [6,29].

This study also provides some evidence for the feasibility of using ASCOT with older people. All 301 participants responded to every item in the instrument, although about ten per cent of the responses were given by proxy. The need for proxies is not unexpected in a sample of older people receiving social care, where prevalence of cognitive decline is likely to be quite high. Although cognitive ability was not something we recorded in this study, the fact that the rate of proxy response was no higher for ASCOT than other QoL, health and disability measures (including the EQ-5D, ADLs and GHQ-12), suggests that proxy responses were given because the respondent lacked the capacity to answer survey questions in general rather than the ASCOT questions specifically. Further work examining the types of people for whom a proxy response is required and whether there are systematic differences between responses given by proxy or by self, as has been found elsewhere [30-33], would be helpful, as would the development of a suitable version for use with proxy respondents.

For the most part, the distribution of the items seemed plausible. Although the distributions were skewed towards good outcomes, if services are doing their job properly this type of distribution is to be expected. However, the distributions for *Food and drink *and *Accommodation *were highly skewed. We, therefore, tested revised wording in a parallel piece of work [34,35]. The new wording achieved better distributions in a sample of equipment users (see Appendix A) and has now been incorporated into the revised measure.

There were some limitations associated with this study. Firstly, the sample data only included older people receiving publicly-funded home care services. As a result it is only possible to draw conclusions about the feasibility of using the measure and its validity for this client group in this setting. Secondly, the sample obtained here was not ethnically diverse, so we cannot demonstrate the validity of the measure amongst black and minority ethnic (BME) groups. It would therefore be of value to repeat this analysis with other client groups and, given the potential for some members of BME groups to have very specific preferences related to their cultural heritage, on a more ethnically diverse sample. Future work should also consider the reliability of the items.

## Conclusion

The current policy emphasis on outcomes in the field of health and social care is unlikely to reduce as fiscal pressures intensify the need to identify value for money. It is important that any measure provide a valid description of the outcomes states it is intended to reflect. The results for ASCOT are encouraging in this respect, although further validation work with a different sample and the development of a version suitable for proxies would be of value.

## Abbreviations

The following abbreviations have been used: ADL: Activity of daily living; ASCOT: Adult Social Care Outcomes Toolkit; BME: Black and minority ethnic; CASP-12: 12 item scale measuring control, autonomy, self-realisation and pleasure; EQ-5D: EuroQol 5-item health questionnaire; GHQ-12: 12-item General Health Questionnaire; IADL: Instrumental activity of daily living; LA: Local authority; NIHR: National Institute for Health Research; SCRQoL: Social Care Related Quality of Life; SEG: Socio-economic group; QoL: Quality of Life; UCLA: University of California, Los Angeles; and UES: user experience survey). In tables 5 and 6 we use Accomm as shorthand for accommodation.

## Competing interests

The authors declare that they have no competing interests.

## Authors' contributions

JM designed this part of the broader study, conducted part of the statistical analysis and drafted the manuscript. A-MT conducted part of the statistical analysis, helped to draft the manuscript and is the corresponding author for submission purposes. AN conceived of the study, and participated in its design and organisation and the drafting of the manuscript. JB, TF and JF contributed to the design of the study and the drafting of the manuscript. All authors have read and approved the final manuscript.

## Appendix A

Revised wording of items after further testing:

### Food and drink

Thinking about the food and drink you get, which of the following statements best describes your situation?

I get all the food and drink I like when I want; I get adequate food and drink at OK times; I don't always get adequate or timely food and drink; I don't always get adequate or timely food and drink, and I think there is a risk to my health

### Accommodation cleanliness and comfort

Which of the following statements best describes how clean and comfortable your home is?

My home is as clean and comfortable as I want; My home is adequately clean and comfortable; My home is not quite clean or comfortable enough; My home is not at all clean or comfortable
